# [2-(Tetra­zol-1-yl)acetato-κ*O*]tris­(tri­phenyl­phosphine-κ*P*)silver(I) mono­hydrate

**DOI:** 10.1107/S1600536809048144

**Published:** 2009-11-18

**Authors:** Jun Zhao, Zong-Zhi Hu, Xue-Gang Zheng, Seik Weng Ng

**Affiliations:** a College of Mechanical & Material Engineering, Functional Materials Research Institute, China Three Gorges University, Yichang 443002, People’s Republic of China; b Lanzhou Institute of Biological Products, Lanzhou 730046, People’s Republic of China; c Department of Chemistry, University of Malaya, 50603 Kuala Lumpur, Malaysia

## Abstract

The Ag^I^ atom in the title compound, [Ag(C_3_H_3_N_4_O_2_)(C_18_H_15_P)_3_]·H_2_O, exists in a distorted tetra­hedral environment. The uncoordinated water mol­ecule forms only one hydrogen bond to the uncoordinated carbonyl O atom.

## Related literature

For the crystal structure of silver tetra­zol-1-yl-acetate, see: Dong *et al.* (2008 ▶).
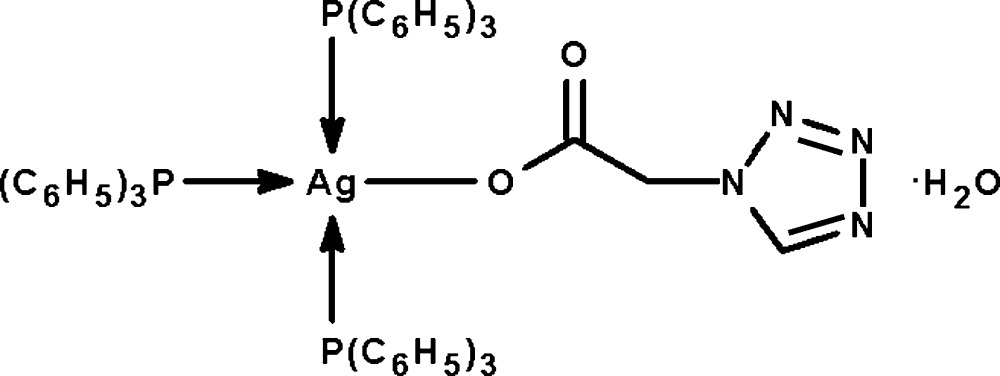


## Experimental

### 

#### Crystal data

[Ag(C_3_H_3_N_4_O_2_)(C_18_H_15_P)_3_]·H_2_O
*M* *_r_* = 1039.79
Monoclinic, 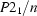

*a* = 13.613 (4) Å
*b* = 23.017 (6) Å
*c* = 16.115 (4) Å
β = 95.590 (3)°
*V* = 5025 (2) Å^3^
*Z* = 4
Mo *K*α radiation
μ = 0.55 mm^−1^
*T* = 293 K
0.40 × 0.20 × 0.20 mm

#### Data collection

Rigaku Mercury CCD diffractometer
Absorption correction: multi-scan (*CrystalClear*; Rigaku, 2002 ▶) *T*_min_ = 0.811, *T*_max_ = 0.899
38977 measured reflections
11514 independent reflections
9437 reflections with *I* > 2σ(*I*)
*R*_int_ = 0.038

#### Refinement

*R*[*F* ^2^ > 2σ(*F* ^2^)] = 0.053
*wR*(*F* ^2^) = 0.139
*S* = 1.06
11514 reflections
505 parameters
6 restraints
H-atom parameters constrained
Δρ_max_ = 0.64 e Å^−3^
Δρ_min_ = −0.55 e Å^−3^

### 

Data collection: *CrystalClear* (Rigaku, 2002 ▶); cell refinement: *CrystalClear*; data reduction: *CrystalClear*; program(s) used to solve structure: *SHELXS97* (Sheldrick, 2008 ▶); program(s) used to refine structure: *SHELXL97* (Sheldrick, 2008 ▶); molecular graphics: *X-SEED* (Barbour, 2001 ▶); software used to prepare material for publication: *publCIF* (Westrip, 2009 ▶).

## Supplementary Material

Crystal structure: contains datablocks global, I. DOI: 10.1107/S1600536809048144/hy2246sup1.cif

Structure factors: contains datablocks I. DOI: 10.1107/S1600536809048144/hy2246Isup2.hkl

Additional supplementary materials:  crystallographic information; 3D view; checkCIF report

## Figures and Tables

**Table 1:** Selected bond lengths (Å)

Ag1—O1	2.360 (3)
Ag1—P1	2.587 (1)
Ag1—P2	2.535 (1)
Ag1—P3	2.496 (1)

**Table 2:** Hydrogen-bond geometry (Å, °)

*D*—H⋯*A*	*D*—H	H⋯*A*	*D*⋯*A*	*D*—H⋯*A*
O1*W*—H1*W*1⋯O2	0.84	2.01	2.800 (8)	157

**Fig. 1.**
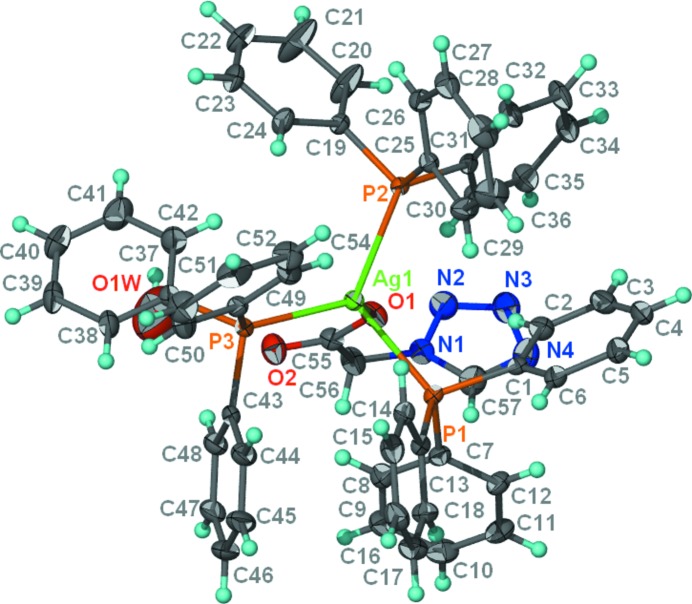
Molecular structure of the title compound. Displacement ellipsoids are drawn at the 30% probability level.

## supplementary crystallographic information

### Experimental

Silver tetrazol-1-yl-acetate (0.024 g, 0.1 mmol) (Dong *et al.*, 2008) and triphenylphosphine (0.079 g, 0.3 mmol) were dissolved in 10 ml dichloromethane. The solution was filtered and set aside for the growth of crystals.

### Refinement

All phenyl rings were refined as rigid hexagons of 1.39 Å sides as there was a slight spead of C—C distances. C-bound H atoms were placed in calculated positions (C—H = 0.93 and 0.97 Å) and were included in the refinement in the riding model approximation, with *U*_iso_(H) = 1.2*U*_eq_(C). The water H atoms were placed in chemically sensible positions (O—H = 0.84 Å) on the basis of hydrogen bonding interactions; the water molecule forms only one hydrogen bond.

A large difference of the components of the anisotropic displacement parameters along the Ag1—P1 and Ag1—P2 bonds was noted. However, there was no contamination of these parameters with other (unresolved) effects such as (substitutional) disorder, model or data errors and/or over-refinement. Neither was the P atoms wrongly assigned, and the multi-scan absorption was adequate.

### Crystal data

**Table:**

[Ag(C_3_H_3_N_4_O_2_)(C_18_H_15_P)_3_]·H_2_O	*F*(000) = 2144
*M**_r_* = 1039.79	*D*_x_ = 1.374 Mg m^−^^3^
Monoclinic, *P*2_1_/*n*	Mo *K*α radiation, λ = 0.71073 Å
Hall symbol: -P 2yn	Cell parameters from 8961 reflections
*a* = 13.613 (4) Å	θ = 2.1–27.5°
*b* = 23.017 (6) Å	µ = 0.55 mm^−^^1^
*c* = 16.115 (4) Å	*T* = 293 K
β = 95.590 (3)°	Block, colorless
*V* = 5025 (2) Å^3^	0.40 × 0.20 × 0.20 mm
*Z* = 4	

### Data collection

**Table:**

Rigaku Mercury CCD diffractometer	11514 independent reflections
Radiation source: fine-focus sealed tube	9437 reflections with *I* > 2σ(*I*)
graphite	*R*_int_ = 0.038
ω scan	θ_max_ = 27.5°, θ_min_ = 2.3°
Absorption correction: multi-scan (*CrystalClear*; Rigaku, 2002)	*h* = −16→17
*T*_min_ = 0.811, *T*_max_ = 0.899	*k* = −29→26
38977 measured reflections	*l* = −20→20

### Refinement

**Table:**

Refinement on *F*^2^	Primary atom site location: structure-invariant direct methods
Least-squares matrix: full	Secondary atom site location: difference Fourier map
*R*[*F*^2^ > 2σ(*F*^2^)] = 0.053	Hydrogen site location: inferred from neighbouring sites
*wR*(*F*^2^) = 0.139	H-atom parameters constrained
*S* = 1.06	*w* = 1/[σ^2^(*F*_o_^2^) + (0.065*P*)^2^ + 3.6165*P*] where *P* = (*F*_o_^2^ + 2*F*_c_^2^)/3
11514 reflections	(Δ/σ)_max_ = 0.001
505 parameters	Δρ_max_ = 0.64 e Å^−^^3^
6 restraints	Δρ_min_ = −0.55 e Å^−^^3^

### Fractional atomic coordinates and isotropic or equivalent isotropic displacement parameters (Å^2^)

**Table:**

	*x*	*y*	*z*	*U*_iso_*/*U*_eq_	
Ag1	0.599944 (17)	0.636548 (11)	0.735961 (14)	0.03902 (9)	
P1	0.72965 (6)	0.67625 (4)	0.84989 (5)	0.0411 (2)	
P2	0.63570 (6)	0.65042 (4)	0.58575 (5)	0.03453 (18)	
P3	0.43100 (6)	0.65097 (4)	0.78029 (5)	0.03721 (19)	
O1	0.6750 (2)	0.54428 (11)	0.75165 (16)	0.0564 (7)	
O2	0.5471 (2)	0.50271 (14)	0.8047 (2)	0.0695 (8)	
O1W	0.3908 (6)	0.4246 (4)	0.7697 (6)	0.267 (4)	
H1W1	0.4273	0.4541	0.7718	0.401*	
H1W2	0.3836	0.4122	0.7204	0.401*	
N1	0.7978 (2)	0.45528 (13)	0.7941 (2)	0.0510 (7)	
N2	0.8329 (3)	0.42544 (19)	0.7329 (2)	0.0766 (11)	
N3	0.9236 (3)	0.4415 (2)	0.7300 (3)	0.0852 (13)	
N4	0.9487 (3)	0.4813 (2)	0.7895 (3)	0.0849 (12)	
C1	0.84036 (14)	0.70195 (11)	0.80516 (14)	0.0445 (8)	
C2	0.8852 (2)	0.66286 (10)	0.75500 (16)	0.0631 (11)	
H2	0.8605	0.6253	0.7477	0.076*	
C3	0.9670 (2)	0.67986 (15)	0.71568 (16)	0.0797 (15)	
H3	0.9971	0.6537	0.6821	0.096*	
C4	1.00398 (16)	0.73593 (16)	0.72653 (17)	0.0792 (16)	
H4	1.0587	0.7473	0.7002	0.095*	
C5	0.95910 (19)	0.77502 (12)	0.77669 (18)	0.0720 (13)	
H5	0.9838	0.8125	0.7839	0.086*	
C6	0.87729 (18)	0.75802 (10)	0.81600 (15)	0.0551 (9)	
H6	0.8473	0.7842	0.8496	0.066*	
C7	0.77831 (16)	0.62218 (10)	0.92662 (13)	0.0448 (8)	
C8	0.71101 (14)	0.58444 (12)	0.95758 (16)	0.0613 (10)	
H8	0.6447	0.5862	0.9375	0.074*	
C9	0.7429 (2)	0.54412 (12)	1.01856 (17)	0.0757 (13)	
H9	0.6978	0.5189	1.0393	0.091*	
C10	0.8420 (2)	0.54154 (11)	1.04857 (16)	0.0742 (13)	
H10	0.8633	0.5146	1.0894	0.089*	
C11	0.90932 (15)	0.57928 (12)	1.01761 (16)	0.0645 (11)	
H11	0.9757	0.5776	1.0377	0.077*	
C12	0.87747 (15)	0.61960 (11)	0.95663 (15)	0.0538 (9)	
H12	0.9225	0.6449	0.9359	0.065*	
C13	0.69582 (17)	0.73678 (9)	0.91528 (12)	0.0425 (7)	
C14	0.64237 (17)	0.78217 (11)	0.87600 (11)	0.0513 (9)	
H14	0.6266	0.7815	0.8185	0.062*	
C15	0.61245 (18)	0.82861 (10)	0.92259 (16)	0.0617 (10)	
H15	0.5767	0.8590	0.8963	0.074*	
C16	0.6360 (2)	0.82967 (10)	1.00846 (16)	0.0655 (11)	
H16	0.6160	0.8608	1.0396	0.079*	
C17	0.6895 (2)	0.78428 (12)	1.04775 (10)	0.0660 (11)	
H17	0.7052	0.7850	1.1052	0.079*	
C18	0.71937 (18)	0.73784 (10)	1.00116 (12)	0.0540 (9)	
H18	0.7551	0.7075	1.0274	0.065*	
C19	0.54054 (14)	0.61888 (11)	0.51130 (15)	0.0410 (7)	
C20	0.56112 (16)	0.57351 (15)	0.4588 (2)	0.114 (3)	
H20	0.6257	0.5605	0.4576	0.137*	
C21	0.4852 (2)	0.54751 (15)	0.4079 (2)	0.140 (3)	
H21	0.4990	0.5172	0.3728	0.168*	
C22	0.38870 (19)	0.56688 (14)	0.4097 (2)	0.0758 (13)	
H22	0.3379	0.5495	0.3756	0.091*	
C23	0.36812 (13)	0.61224 (14)	0.46219 (19)	0.0696 (12)	
H23	0.3036	0.6252	0.4633	0.083*	
C24	0.44404 (16)	0.63825 (12)	0.51302 (17)	0.0608 (11)	
H24	0.4303	0.6686	0.5482	0.073*	
C25	0.64115 (17)	0.72619 (8)	0.55331 (13)	0.0376 (7)	
C26	0.60325 (17)	0.74622 (10)	0.47532 (12)	0.0481 (8)	
H26	0.5697	0.7209	0.4375	0.058*	
C27	0.6155 (2)	0.80408 (11)	0.45384 (14)	0.0607 (10)	
H27	0.5901	0.8175	0.4017	0.073*	
C28	0.6656 (2)	0.84190 (8)	0.5104 (2)	0.0786 (14)	
H28	0.6738	0.8806	0.4960	0.094*	
C29	0.7035 (2)	0.82187 (10)	0.58835 (17)	0.0884 (16)	
H29	0.7371	0.8472	0.6262	0.106*	
C30	0.6913 (2)	0.76401 (11)	0.60982 (12)	0.0614 (11)	
H30	0.7167	0.7506	0.6620	0.074*	
C31	0.74926 (14)	0.62037 (10)	0.55056 (14)	0.0395 (7)	
C32	0.79144 (18)	0.64384 (10)	0.48291 (15)	0.0627 (11)	
H32	0.7639	0.6766	0.4562	0.075*	
C33	0.87483 (19)	0.61828 (13)	0.45522 (16)	0.0728 (13)	
H33	0.9031	0.6340	0.4100	0.087*	
C34	0.91602 (16)	0.56926 (12)	0.49519 (17)	0.0644 (11)	
H34	0.9718	0.5522	0.4767	0.077*	
C35	0.87383 (18)	0.54580 (10)	0.56285 (16)	0.0617 (10)	
H35	0.9014	0.5130	0.5896	0.074*	
C36	0.79045 (17)	0.57135 (10)	0.59053 (13)	0.0485 (8)	
H36	0.7622	0.5557	0.6358	0.058*	
C37	0.32834 (15)	0.60244 (11)	0.74617 (15)	0.0423 (7)	
C38	0.24374 (18)	0.59950 (13)	0.78783 (15)	0.0623 (11)	
H38	0.2389	0.6217	0.8355	0.075*	
C39	0.16630 (15)	0.56336 (14)	0.7583 (2)	0.0726 (13)	
H39	0.1097	0.5614	0.7862	0.087*	
C40	0.1735 (2)	0.53015 (13)	0.6871 (2)	0.0822 (15)	
H40	0.1217	0.5060	0.6673	0.099*	
C41	0.2581 (2)	0.53309 (14)	0.64542 (18)	0.0932 (17)	
H41	0.2629	0.5109	0.5978	0.112*	
C42	0.33550 (18)	0.56923 (13)	0.67497 (16)	0.0685 (12)	
H42	0.3921	0.5712	0.6471	0.082*	
C43	0.43479 (18)	0.64740 (10)	0.89372 (10)	0.0437 (8)	
C44	0.4426 (2)	0.69731 (9)	0.94247 (15)	0.0633 (11)	
H44	0.4379	0.7337	0.9173	0.076*	
C45	0.4573 (2)	0.69284 (11)	1.02880 (14)	0.0846 (17)	
H45	0.4625	0.7262	1.0614	0.102*	
C46	0.4643 (2)	0.63845 (14)	1.06639 (10)	0.0809 (16)	
H46	0.4741	0.6355	1.1242	0.097*	
C47	0.4565 (2)	0.58853 (11)	1.01765 (15)	0.0673 (12)	
H47	0.4611	0.5521	1.0428	0.081*	
C48	0.44175 (19)	0.59300 (9)	0.93131 (14)	0.0544 (9)	
H48	0.4365	0.5596	0.8987	0.065*	
C49	0.38348 (17)	0.72416 (8)	0.75446 (15)	0.0419 (7)	
C50	0.29639 (17)	0.74544 (11)	0.78129 (17)	0.0632 (11)	
H50	0.2588	0.7221	0.8132	0.076*	
C51	0.26542 (18)	0.80157 (12)	0.76044 (19)	0.0775 (14)	
H51	0.2072	0.8158	0.7784	0.093*	
C52	0.3215 (2)	0.83642 (9)	0.7128 (2)	0.0777 (14)	
H52	0.3008	0.8740	0.6988	0.093*	
C53	0.4086 (2)	0.81515 (10)	0.68593 (18)	0.0726 (12)	
H53	0.4462	0.8385	0.6540	0.087*	
C54	0.43960 (16)	0.75902 (11)	0.70678 (15)	0.0541 (9)	
H54	0.4979	0.7448	0.6888	0.065*	
C55	0.6329 (3)	0.50491 (16)	0.7867 (2)	0.0462 (8)	
C56	0.6954 (3)	0.45096 (17)	0.8125 (3)	0.0581 (10)	
H56A	0.6940	0.4448	0.8720	0.070*	
H56B	0.6656	0.4173	0.7840	0.070*	
C57	0.8694 (4)	0.4888 (2)	0.8271 (3)	0.0757 (13)	
H57	0.8641	0.5143	0.8711	0.091*	

### Atomic displacement parameters (Å^2^)

**Table:**

	*U*^11^	*U*^22^	*U*^33^	*U*^12^	*U*^13^	*U*^23^
Ag1	0.03652 (14)	0.04310 (16)	0.03750 (14)	0.00269 (10)	0.00390 (9)	0.00264 (10)
P1	0.0354 (4)	0.0509 (5)	0.0357 (4)	−0.0027 (4)	−0.0027 (3)	−0.0002 (4)
P2	0.0310 (4)	0.0413 (5)	0.0313 (4)	−0.0004 (3)	0.0032 (3)	−0.0013 (3)
P3	0.0334 (4)	0.0447 (5)	0.0342 (4)	−0.0020 (3)	0.0066 (3)	0.0015 (3)
O1	0.0673 (17)	0.0433 (15)	0.0611 (16)	0.0095 (12)	0.0191 (13)	0.0133 (12)
O2	0.0553 (17)	0.069 (2)	0.087 (2)	0.0087 (14)	0.0180 (15)	0.0076 (16)
O1W	0.250 (7)	0.252 (8)	0.285 (8)	−0.105 (6)	−0.045 (6)	0.096 (6)
N1	0.0535 (18)	0.0413 (17)	0.0586 (18)	0.0103 (14)	0.0069 (14)	0.0084 (14)
N2	0.068 (2)	0.088 (3)	0.075 (2)	0.010 (2)	0.0119 (19)	−0.022 (2)
N3	0.060 (2)	0.111 (4)	0.086 (3)	0.017 (2)	0.015 (2)	−0.007 (3)
N4	0.057 (2)	0.093 (3)	0.104 (3)	0.005 (2)	0.006 (2)	−0.001 (3)
C1	0.0352 (16)	0.066 (2)	0.0310 (15)	−0.0001 (15)	−0.0027 (13)	0.0012 (15)
C2	0.057 (2)	0.082 (3)	0.051 (2)	0.001 (2)	0.0082 (18)	−0.008 (2)
C3	0.059 (3)	0.132 (5)	0.050 (2)	0.012 (3)	0.015 (2)	0.000 (3)
C4	0.045 (2)	0.143 (5)	0.050 (2)	−0.002 (3)	0.0074 (18)	0.026 (3)
C5	0.055 (2)	0.098 (4)	0.062 (3)	−0.023 (2)	0.000 (2)	0.023 (2)
C6	0.050 (2)	0.070 (3)	0.0447 (19)	−0.0103 (18)	−0.0005 (16)	0.0071 (18)
C7	0.0456 (18)	0.049 (2)	0.0388 (17)	−0.0014 (15)	−0.0008 (14)	−0.0041 (14)
C8	0.056 (2)	0.070 (3)	0.057 (2)	−0.008 (2)	0.0052 (18)	0.009 (2)
C9	0.090 (3)	0.073 (3)	0.065 (3)	−0.022 (3)	0.012 (2)	0.017 (2)
C10	0.100 (4)	0.063 (3)	0.057 (2)	0.004 (3)	−0.002 (2)	0.017 (2)
C11	0.066 (3)	0.064 (3)	0.059 (2)	0.008 (2)	−0.017 (2)	0.005 (2)
C12	0.047 (2)	0.058 (2)	0.054 (2)	−0.0012 (17)	−0.0085 (16)	0.0022 (18)
C13	0.0351 (16)	0.053 (2)	0.0387 (16)	−0.0029 (14)	0.0009 (13)	0.0011 (14)
C14	0.0386 (18)	0.064 (2)	0.051 (2)	0.0021 (16)	0.0020 (15)	0.0049 (17)
C15	0.048 (2)	0.060 (3)	0.079 (3)	0.0116 (18)	0.0122 (19)	0.006 (2)
C16	0.066 (3)	0.063 (3)	0.071 (3)	−0.001 (2)	0.027 (2)	−0.012 (2)
C17	0.080 (3)	0.072 (3)	0.048 (2)	0.000 (2)	0.011 (2)	−0.009 (2)
C18	0.059 (2)	0.061 (3)	0.0411 (18)	0.0051 (18)	−0.0004 (16)	0.0005 (17)
C19	0.0357 (16)	0.0454 (19)	0.0411 (17)	−0.0010 (13)	−0.0001 (13)	−0.0018 (14)
C20	0.058 (3)	0.129 (5)	0.146 (5)	0.026 (3)	−0.032 (3)	−0.095 (4)
C21	0.083 (4)	0.142 (6)	0.181 (7)	0.031 (4)	−0.058 (4)	−0.116 (6)
C22	0.065 (3)	0.078 (3)	0.078 (3)	−0.010 (2)	−0.026 (2)	−0.016 (3)
C23	0.0359 (19)	0.098 (4)	0.073 (3)	−0.003 (2)	−0.0028 (18)	−0.003 (3)
C24	0.0374 (19)	0.075 (3)	0.068 (3)	0.0053 (18)	−0.0028 (17)	−0.019 (2)
C25	0.0378 (16)	0.0366 (17)	0.0392 (16)	0.0006 (13)	0.0078 (13)	−0.0010 (13)
C26	0.0472 (19)	0.049 (2)	0.0476 (19)	−0.0015 (15)	−0.0001 (15)	0.0076 (16)
C27	0.058 (2)	0.057 (3)	0.068 (3)	0.0069 (19)	0.0126 (19)	0.024 (2)
C28	0.094 (4)	0.046 (3)	0.099 (4)	−0.002 (2)	0.028 (3)	0.014 (2)
C29	0.128 (5)	0.053 (3)	0.083 (3)	−0.033 (3)	0.006 (3)	−0.014 (3)
C30	0.080 (3)	0.051 (2)	0.051 (2)	−0.017 (2)	0.0000 (19)	−0.0073 (18)
C31	0.0297 (14)	0.049 (2)	0.0399 (16)	0.0007 (13)	0.0025 (12)	−0.0042 (14)
C32	0.049 (2)	0.077 (3)	0.065 (3)	0.0173 (19)	0.0231 (19)	0.024 (2)
C33	0.051 (2)	0.092 (4)	0.081 (3)	0.014 (2)	0.035 (2)	0.019 (3)
C34	0.045 (2)	0.075 (3)	0.076 (3)	0.0121 (19)	0.0194 (19)	−0.002 (2)
C35	0.056 (2)	0.058 (3)	0.072 (3)	0.0178 (19)	0.012 (2)	0.006 (2)
C36	0.0493 (19)	0.050 (2)	0.0469 (19)	0.0073 (16)	0.0108 (15)	0.0024 (16)
C37	0.0399 (17)	0.045 (2)	0.0423 (17)	−0.0017 (14)	0.0040 (13)	0.0015 (14)
C38	0.044 (2)	0.076 (3)	0.068 (3)	−0.0125 (19)	0.0167 (18)	−0.009 (2)
C39	0.042 (2)	0.079 (3)	0.096 (3)	−0.014 (2)	0.008 (2)	0.008 (3)
C40	0.063 (3)	0.072 (3)	0.107 (4)	−0.017 (2)	−0.014 (3)	−0.014 (3)
C41	0.086 (4)	0.089 (4)	0.103 (4)	−0.022 (3)	−0.001 (3)	−0.043 (3)
C42	0.063 (3)	0.073 (3)	0.071 (3)	−0.008 (2)	0.014 (2)	−0.019 (2)
C43	0.0335 (16)	0.062 (2)	0.0363 (16)	−0.0066 (14)	0.0085 (13)	−0.0013 (15)
C44	0.075 (3)	0.069 (3)	0.048 (2)	−0.025 (2)	0.0131 (19)	−0.0057 (19)
C45	0.104 (4)	0.109 (4)	0.043 (2)	−0.046 (3)	0.016 (2)	−0.015 (2)
C46	0.083 (3)	0.123 (5)	0.036 (2)	−0.028 (3)	0.007 (2)	0.005 (2)
C47	0.061 (2)	0.095 (4)	0.047 (2)	0.005 (2)	0.0117 (18)	0.021 (2)
C48	0.053 (2)	0.066 (3)	0.0447 (19)	0.0034 (18)	0.0112 (16)	0.0062 (17)
C49	0.0404 (17)	0.045 (2)	0.0404 (17)	0.0014 (14)	0.0017 (13)	0.0002 (14)
C50	0.054 (2)	0.057 (3)	0.080 (3)	0.0036 (19)	0.016 (2)	0.000 (2)
C51	0.071 (3)	0.068 (3)	0.093 (3)	0.026 (2)	0.006 (3)	−0.011 (3)
C52	0.100 (4)	0.048 (3)	0.080 (3)	0.019 (2)	−0.014 (3)	0.001 (2)
C53	0.087 (3)	0.055 (3)	0.075 (3)	0.003 (2)	0.007 (2)	0.020 (2)
C54	0.056 (2)	0.055 (2)	0.052 (2)	−0.0014 (17)	0.0054 (17)	0.0058 (17)
C55	0.053 (2)	0.0399 (19)	0.0466 (19)	0.0054 (15)	0.0078 (15)	−0.0007 (15)
C56	0.058 (2)	0.047 (2)	0.072 (3)	0.0082 (17)	0.0182 (19)	0.0155 (19)
C57	0.068 (3)	0.075 (3)	0.084 (3)	0.001 (2)	0.007 (2)	−0.020 (3)

### Geometric parameters (Å, °)

**Table:**

Ag1—O1	2.360 (3)	C22—H22	0.9300
Ag1—P1	2.587 (1)	C23—C24	1.3900
Ag1—P2	2.535 (1)	C23—H23	0.9300
Ag1—P3	2.496 (1)	C24—H24	0.9300
P1—C1	1.8309 (19)	C25—C26	1.3900
P1—C7	1.832 (2)	C25—C30	1.3900
P1—C13	1.8325 (19)	C26—C27	1.3900
P2—C25	1.8243 (19)	C26—H26	0.9300
P2—C19	1.8280 (19)	C27—C28	1.3900
P2—C31	1.8330 (17)	C27—H27	0.9300
P3—C43	1.8255 (19)	C28—C29	1.3900
P3—C37	1.831 (2)	C28—H28	0.9300
P3—C49	1.838 (2)	C29—C30	1.3900
O1—C55	1.238 (4)	C29—H29	0.9300
O2—C55	1.232 (4)	C30—H30	0.9300
O1W—H1W1	0.8401	C31—C32	1.3900
O1W—H1W2	0.8400	C31—C36	1.3900
N1—C57	1.314 (6)	C32—C33	1.3900
N1—N2	1.328 (5)	C32—H32	0.9300
N1—C56	1.457 (5)	C33—C34	1.3900
N2—N3	1.294 (6)	C33—H33	0.9300
N3—N4	1.345 (6)	C34—C35	1.3900
N4—C57	1.299 (6)	C34—H34	0.9300
C1—C2	1.3900	C35—C36	1.3900
C1—C6	1.3900	C35—H35	0.9300
C2—C3	1.3900	C36—H36	0.9300
C2—H2	0.9300	C37—C38	1.3900
C3—C4	1.3900	C37—C42	1.3900
C3—H3	0.9300	C38—C39	1.3900
C4—C5	1.3900	C38—H38	0.9300
C4—H4	0.9300	C39—C40	1.3900
C5—C6	1.3900	C39—H39	0.9300
C5—H5	0.9300	C40—C41	1.3900
C6—H6	0.9300	C40—H40	0.9300
C7—C8	1.3900	C41—C42	1.3900
C7—C12	1.3900	C41—H41	0.9300
C8—C9	1.3900	C42—H42	0.9300
C8—H8	0.9300	C43—C44	1.3900
C9—C10	1.3900	C43—C48	1.3900
C9—H9	0.9300	C44—C45	1.3900
C10—C11	1.3900	C44—H44	0.9300
C10—H10	0.9300	C45—C46	1.3900
C11—C12	1.3900	C45—H45	0.9300
C11—H11	0.9300	C46—C47	1.3900
C12—H12	0.9300	C46—H46	0.9300
C13—C14	1.3900	C47—C48	1.3900
C13—C18	1.3900	C47—H47	0.9300
C14—C15	1.3900	C48—H48	0.9300
C14—H14	0.9300	C49—C50	1.3900
C15—C16	1.3900	C49—C54	1.3900
C15—H15	0.9300	C50—C51	1.3900
C16—C17	1.3900	C50—H50	0.9300
C16—H16	0.9300	C51—C52	1.3900
C17—C18	1.3900	C51—H51	0.9300
C17—H17	0.9300	C52—C53	1.3900
C18—H18	0.9300	C52—H52	0.9300
C19—C20	1.3900	C53—C54	1.3900
C19—C24	1.3900	C53—H53	0.9300
C20—C21	1.3900	C54—H54	0.9300
C20—H20	0.9300	C55—C56	1.540 (5)
C21—C22	1.3900	C56—H56A	0.9700
C21—H21	0.9300	C56—H56B	0.9700
C22—C23	1.3900	C57—H57	0.9300
			
O1—Ag1—P1	89.04 (8)	C19—C24—H24	120.0
O1—Ag1—P2	95.40 (6)	C26—C25—C30	120.0
O1—Ag1—P3	119.38 (7)	C26—C25—P2	123.67 (13)
P1—Ag1—P2	116.87 (3)	C30—C25—P2	116.23 (13)
P1—Ag1—P3	109.52 (3)	C27—C26—C25	120.0
P2—Ag1—P3	121.63 (3)	C27—C26—H26	120.0
C1—P1—C7	103.17 (11)	C25—C26—H26	120.0
C1—P1—C13	104.08 (12)	C28—C27—C26	120.0
C7—P1—C13	102.88 (11)	C28—C27—H27	120.0
C1—P1—Ag1	111.46 (8)	C26—C27—H27	120.0
C7—P1—Ag1	114.58 (9)	C27—C28—C29	120.0
C13—P1—Ag1	118.97 (8)	C27—C28—H28	120.0
C25—P2—C19	103.75 (12)	C29—C28—H28	120.0
C25—P2—C31	102.30 (11)	C28—C29—C30	120.0
C19—P2—C31	102.25 (11)	C28—C29—H29	120.0
C25—P2—Ag1	114.26 (8)	C30—C29—H29	120.0
C19—P2—Ag1	112.76 (9)	C29—C30—C25	120.0
C31—P2—Ag1	119.60 (8)	C29—C30—H30	120.0
C43—P3—C37	102.68 (11)	C25—C30—H30	120.0
C43—P3—C49	104.07 (12)	C32—C31—C36	120.0
C37—P3—C49	104.46 (12)	C32—C31—P2	121.42 (13)
C43—P3—Ag1	110.10 (9)	C36—C31—P2	118.50 (13)
C37—P3—Ag1	122.03 (9)	C33—C32—C31	120.0
C49—P3—Ag1	111.77 (8)	C33—C32—H32	120.0
C55—O1—Ag1	119.5 (2)	C31—C32—H32	120.0
H1W1—O1W—H1W2	109.0	C32—C33—C34	120.0
C57—N1—N2	107.3 (4)	C32—C33—H33	120.0
C57—N1—C56	130.2 (4)	C34—C33—H33	120.0
N2—N1—C56	122.5 (4)	C35—C34—C33	120.0
N3—N2—N1	107.0 (4)	C35—C34—H34	120.0
N2—N3—N4	110.3 (4)	C33—C34—H34	120.0
C57—N4—N3	104.9 (4)	C34—C35—C36	120.0
C2—C1—C6	120.0	C34—C35—H35	120.0
C2—C1—P1	116.43 (14)	C36—C35—H35	120.0
C6—C1—P1	123.51 (14)	C35—C36—C31	120.0
C1—C2—C3	120.0	C35—C36—H36	120.0
C1—C2—H2	120.0	C31—C36—H36	120.0
C3—C2—H2	120.0	C38—C37—C42	120.0
C4—C3—C2	120.0	C38—C37—P3	121.91 (14)
C4—C3—H3	120.0	C42—C37—P3	118.06 (14)
C2—C3—H3	120.0	C37—C38—C39	120.0
C3—C4—C5	120.0	C37—C38—H38	120.0
C3—C4—H4	120.0	C39—C38—H38	120.0
C5—C4—H4	120.0	C40—C39—C38	120.0
C6—C5—C4	120.0	C40—C39—H39	120.0
C6—C5—H5	120.0	C38—C39—H39	120.0
C4—C5—H5	120.0	C39—C40—C41	120.0
C5—C6—C1	120.0	C39—C40—H40	120.0
C5—C6—H6	120.0	C41—C40—H40	120.0
C1—C6—H6	120.0	C42—C41—C40	120.0
C8—C7—C12	120.0	C42—C41—H41	120.0
C8—C7—P1	117.33 (14)	C40—C41—H41	120.0
C12—C7—P1	122.61 (14)	C41—C42—C37	120.0
C9—C8—C7	120.0	C41—C42—H42	120.0
C9—C8—H8	120.0	C37—C42—H42	120.0
C7—C8—H8	120.0	C44—C43—C48	120.0
C10—C9—C8	120.0	C44—C43—P3	121.45 (14)
C10—C9—H9	120.0	C48—C43—P3	118.10 (14)
C8—C9—H9	120.0	C45—C44—C43	120.0
C11—C10—C9	120.0	C45—C44—H44	120.0
C11—C10—H10	120.0	C43—C44—H44	120.0
C9—C10—H10	120.0	C44—C45—C46	120.0
C10—C11—C12	120.0	C44—C45—H45	120.0
C10—C11—H11	120.0	C46—C45—H45	120.0
C12—C11—H11	120.0	C47—C46—C45	120.0
C11—C12—C7	120.0	C47—C46—H46	120.0
C11—C12—H12	120.0	C45—C46—H46	120.0
C7—C12—H12	120.0	C48—C47—C46	120.0
C14—C13—C18	120.0	C48—C47—H47	120.0
C14—C13—P1	117.34 (13)	C46—C47—H47	120.0
C18—C13—P1	122.64 (13)	C47—C48—C43	120.0
C15—C14—C13	120.0	C47—C48—H48	120.0
C15—C14—H14	120.0	C43—C48—H48	120.0
C13—C14—H14	120.0	C50—C49—C54	120.0
C16—C15—C14	120.0	C50—C49—P3	123.00 (14)
C16—C15—H15	120.0	C54—C49—P3	117.00 (14)
C14—C15—H15	120.0	C49—C50—C51	120.0
C15—C16—C17	120.0	C49—C50—H50	120.0
C15—C16—H16	120.0	C51—C50—H50	120.0
C17—C16—H16	120.0	C52—C51—C50	120.0
C16—C17—C18	120.0	C52—C51—H51	120.0
C16—C17—H17	120.0	C50—C51—H51	120.0
C18—C17—H17	120.0	C51—C52—C53	120.0
C17—C18—C13	120.0	C51—C52—H52	120.0
C17—C18—H18	120.0	C53—C52—H52	120.0
C13—C18—H18	120.0	C52—C53—C54	120.0
C20—C19—C24	120.0	C52—C53—H53	120.0
C20—C19—P2	121.75 (14)	C54—C53—H53	120.0
C24—C19—P2	118.05 (14)	C53—C54—C49	120.0
C21—C20—C19	120.0	C53—C54—H54	120.0
C21—C20—H20	120.0	C49—C54—H54	120.0
C19—C20—H20	120.0	O2—C55—O1	129.1 (4)
C20—C21—C22	120.0	O2—C55—C56	114.5 (3)
C20—C21—H21	120.0	O1—C55—C56	116.4 (3)
C22—C21—H21	120.0	N1—C56—C55	113.9 (3)
C21—C22—C23	120.0	N1—C56—H56A	108.8
C21—C22—H22	120.0	C55—C56—H56A	108.8
C23—C22—H22	120.0	N1—C56—H56B	108.8
C22—C23—C24	120.0	C55—C56—H56B	108.8
C22—C23—H23	120.0	H56A—C56—H56B	107.7
C24—C23—H23	120.0	N4—C57—N1	110.6 (4)
C23—C24—C19	120.0	N4—C57—H57	124.7
C23—C24—H24	120.0	N1—C57—H57	124.7
			
O1—Ag1—P1—C1	88.43 (11)	C20—C21—C22—C23	0.0
P3—Ag1—P1—C1	−150.59 (10)	C21—C22—C23—C24	0.0
P2—Ag1—P1—C1	−7.14 (10)	C22—C23—C24—C19	0.0
O1—Ag1—P1—C7	−28.24 (11)	C20—C19—C24—C23	0.0
P3—Ag1—P1—C7	92.73 (10)	P2—C19—C24—C23	−174.9 (2)
P2—Ag1—P1—C7	−123.82 (9)	C19—P2—C25—C26	−17.68 (18)
O1—Ag1—P1—C13	−150.48 (11)	C31—P2—C25—C26	88.40 (17)
P3—Ag1—P1—C13	−29.50 (10)	Ag1—P2—C25—C26	−140.85 (13)
P2—Ag1—P1—C13	113.95 (10)	C19—P2—C25—C30	165.90 (15)
O1—Ag1—P2—C25	−155.08 (11)	C31—P2—C25—C30	−88.02 (16)
P3—Ag1—P2—C25	75.35 (9)	Ag1—P2—C25—C30	42.73 (15)
P1—Ag1—P2—C25	−63.41 (9)	C30—C25—C26—C27	0.0
O1—Ag1—P2—C19	86.78 (12)	P2—C25—C26—C27	−176.29 (19)
P3—Ag1—P2—C19	−42.80 (10)	C25—C26—C27—C28	0.0
P1—Ag1—P2—C19	178.44 (9)	C26—C27—C28—C29	0.0
O1—Ag1—P2—C31	−33.42 (12)	C27—C28—C29—C30	0.0
P3—Ag1—P2—C31	−163.00 (10)	C28—C29—C30—C25	0.0
P1—Ag1—P2—C31	58.24 (11)	C26—C25—C30—C29	0.0
O1—Ag1—P3—C43	69.53 (12)	P2—C25—C30—C29	176.56 (18)
P2—Ag1—P3—C43	−172.19 (9)	C25—P2—C31—C32	−27.63 (17)
P1—Ag1—P3—C43	−30.79 (9)	C19—P2—C31—C32	79.60 (18)
O1—Ag1—P3—C37	−50.78 (14)	Ag1—P2—C31—C32	−155.04 (13)
P2—Ag1—P3—C37	67.50 (12)	C25—P2—C31—C36	155.51 (15)
P1—Ag1—P3—C37	−151.10 (11)	C19—P2—C31—C36	−97.26 (17)
O1—Ag1—P3—C49	−175.32 (12)	Ag1—P2—C31—C36	28.09 (18)
P2—Ag1—P3—C49	−57.04 (10)	C36—C31—C32—C33	0.0
P1—Ag1—P3—C49	84.35 (10)	P2—C31—C32—C33	−176.81 (19)
P3—Ag1—O1—C55	−4.7 (3)	C31—C32—C33—C34	0.0
P2—Ag1—O1—C55	−135.9 (3)	C32—C33—C34—C35	0.0
P1—Ag1—O1—C55	107.2 (3)	C33—C34—C35—C36	0.0
C57—N1—N2—N3	−0.3 (5)	C34—C35—C36—C31	0.0
C56—N1—N2—N3	176.3 (4)	C32—C31—C36—C35	0.0
N1—N2—N3—N4	0.7 (6)	P2—C31—C36—C35	176.91 (19)
N2—N3—N4—C57	−0.8 (6)	C43—P3—C37—C38	37.41 (19)
C7—P1—C1—C2	69.95 (15)	C49—P3—C37—C38	−70.97 (19)
C13—P1—C1—C2	177.09 (13)	Ag1—P3—C37—C38	161.20 (13)
Ag1—P1—C1—C2	−53.49 (14)	C43—P3—C37—C42	−144.53 (17)
C7—P1—C1—C6	−113.02 (17)	C49—P3—C37—C42	107.09 (18)
C13—P1—C1—C6	−5.89 (17)	Ag1—P3—C37—C42	−20.7 (2)
Ag1—P1—C1—C6	123.54 (13)	C42—C37—C38—C39	0.0
C6—C1—C2—C3	0.0	P3—C37—C38—C39	178.0 (2)
P1—C1—C2—C3	177.14 (17)	C37—C38—C39—C40	0.0
C1—C2—C3—C4	0.0	C38—C39—C40—C41	0.0
C2—C3—C4—C5	0.0	C39—C40—C41—C42	0.0
C3—C4—C5—C6	0.0	C40—C41—C42—C37	0.0
C4—C5—C6—C1	0.0	C38—C37—C42—C41	0.0
C2—C1—C6—C5	0.0	P3—C37—C42—C41	−178.1 (2)
P1—C1—C6—C5	−176.93 (19)	C37—P3—C43—C44	−133.54 (16)
C1—P1—C7—C8	−164.93 (15)	C49—P3—C43—C44	−24.87 (18)
C13—P1—C7—C8	87.03 (16)	Ag1—P3—C43—C44	95.06 (15)
Ag1—P1—C7—C8	−43.59 (16)	C37—P3—C43—C48	54.13 (17)
C1—P1—C7—C12	17.90 (18)	C49—P3—C43—C48	162.80 (14)
C13—P1—C7—C12	−90.15 (17)	Ag1—P3—C43—C48	−77.27 (14)
Ag1—P1—C7—C12	139.24 (13)	C48—C43—C44—C45	0.0
C12—C7—C8—C9	0.0	P3—C43—C44—C45	−172.2 (2)
P1—C7—C8—C9	−177.26 (19)	C43—C44—C45—C46	0.0
C7—C8—C9—C10	0.0	C44—C45—C46—C47	0.0
C8—C9—C10—C11	0.0	C45—C46—C47—C48	0.0
C9—C10—C11—C12	0.0	C46—C47—C48—C43	0.0
C10—C11—C12—C7	0.0	C44—C43—C48—C47	0.0
C8—C7—C12—C11	0.0	P3—C43—C48—C47	172.45 (19)
P1—C7—C12—C11	177.1 (2)	C43—P3—C49—C50	−53.29 (18)
C1—P1—C13—C14	82.03 (16)	C37—P3—C49—C50	54.06 (17)
C7—P1—C13—C14	−170.62 (14)	Ag1—P3—C49—C50	−172.09 (13)
Ag1—P1—C13—C14	−42.71 (16)	C43—P3—C49—C54	125.82 (16)
C1—P1—C13—C18	−99.60 (16)	C37—P3—C49—C54	−126.83 (15)
C7—P1—C13—C18	7.75 (17)	Ag1—P3—C49—C54	7.02 (17)
Ag1—P1—C13—C18	135.65 (12)	C54—C49—C50—C51	0.0
C18—C13—C14—C15	0.0	P3—C49—C50—C51	179.1 (2)
P1—C13—C14—C15	178.41 (18)	C49—C50—C51—C52	0.0
C13—C14—C15—C16	0.0	C50—C51—C52—C53	0.0
C14—C15—C16—C17	0.0	C51—C52—C53—C54	0.0
C15—C16—C17—C18	0.0	C52—C53—C54—C49	0.0
C16—C17—C18—C13	0.0	C50—C49—C54—C53	0.0
C14—C13—C18—C17	0.0	P3—C49—C54—C53	−179.14 (19)
P1—C13—C18—C17	−178.32 (19)	Ag1—O1—C55—O2	12.9 (6)
C25—P2—C19—C20	119.2 (2)	Ag1—O1—C55—C56	−166.5 (3)
C31—P2—C19—C20	13.0 (2)	C57—N1—C56—C55	68.5 (6)
Ag1—P2—C19—C20	−116.69 (18)	N2—N1—C56—C55	−107.2 (4)
C25—P2—C19—C24	−66.1 (2)	O2—C55—C56—N1	−177.5 (3)
C31—P2—C19—C24	−172.18 (18)	O1—C55—C56—N1	2.1 (5)
Ag1—P2—C19—C24	58.1 (2)	N3—N4—C57—N1	0.6 (6)
C24—C19—C20—C21	0.0	N2—N1—C57—N4	−0.2 (6)
P2—C19—C20—C21	174.7 (2)	C56—N1—C57—N4	−176.5 (4)
C19—C20—C21—C22	0.0		

### Hydrogen-bond geometry (Å, °)

**Table:**

*D*—H···*A*	*D*—H	H···*A*	*D*···*A*	*D*—H···*A*
O1W—H1W1···O2	0.84	2.01	2.800 (8)	157
